# Use of durvalumab in stage III non‐small‐cell lung cancer based on eligibility for the PACIFIC study

**DOI:** 10.1111/1759-7714.14780

**Published:** 2023-01-10

**Authors:** Emma Boys, Bo Gao, Rina Hui, Inês da Silva, Eric Hau, Harriet Gee, Adnan Nagrial

**Affiliations:** ^1^ Department of Medical Oncology Crown Princess Mary Cancer Centre Westmead New South Wales Australia; ^2^ Blacktown Cancer and Haematology Centre Blacktown Hospital Blacktown New South Wales Australia; ^3^ Sydney Medical School The University of Sydney Sydney New South Wales Australia

**Keywords:** chemoradiotherapy, clinical trial eligibility, consolidation therapy, real‐world population, treatment utilization

## Abstract

**Background:**

Durvalumab following concurrent chemoradiotherapy is standard treatment for unresectable stage III non‐small‐cell lung cancer based on the results of the PACIFIC trial. Based on trial criteria, not all patients are eligible for durvalumab in routine clinical practice.

**Methods:**

We evaluated eligibility for durvalumab in a real‐world clinical setting and the impact of eligibility on outcomes. Consecutive patients treated with concurrent chemoradiotherapy at two tertiary centers between January 2015 and June 2022 were assessed. Clinical characteristics and outcomes were evaluated based on eligibility criteria for the PACIFIC trial.

**Results:**

A total of 126 patients were included. Seventy patients (56%) were eligible for durvalumab. Ineligibility was associated with shorter progression‐free survival of 9.7 months versus 18.4 months (hazard ratio [HR] 0.61, 95% confidence interval [CI] 0.39–0.95, *p* = 0.029) and overall survival of 26.4 months versus 58.7 months (HR 0.47, 95% CI 0.28–0.80*, p* = 0.005). Common reasons for ineligibility were history of previous malignancy (32%) and progressive disease or death during chemoradiotherapy (25%). Ineligible patients who received durvalumab had similar outcomes to eligible patients who received durvalumab.

**Conclusions:**

In a real‐world cohort, adjuvant durvalumab is safe and beneficial in a substantial proportion of patients who would not have been eligible for the PACIFIC trial.

## Graphical abstract

We focused on a cohort of 126 patients with stage III non‐small‐cell lung cancer and evaluated eligibility for the PACIFIC study and durvalumab on outcomes. A large proportion of our cohort were ineligible for the PACIFIC study (44%). Trial eligibility led to improved outcome regardless of receipt of durvalumab. However, durvalumab was also safe and beneficial in many patients who were ineligible for the PACIFIC trial. This data provides further evidence that durvalumab use is safe and beneficial in a real‐world setting.

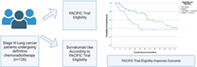

## INTRODUCTION

Lung cancer remains the primary cause of cancer‐related death globally.^1^ Stage III non‐small‐cell lung cancer (NSCLC) is a heterogenous group that accounts for approximately one‐third of all NSCLC cases at diagnosis. The historical standard of care, concurrent chemoradiation, results in 5‐year survival of only 15–30%.^2^ The PACIFIC trial, a randomized, double‐blinded, placebo‐controlled phase 3 trial, investigated the role of 12 months of consolidation durvalumab following concurrent chemoradiation in 713 patients with unresectable stage III NSCLC.^3^ At a median of 34.2 months of follow‐up, progression‐free survival (PFS) was 16.9 months and overall survival (OS) was 47.5 months for patients who received consolidation durvalumab compared to 5.6 and 29.1 months with concurrent chemoradiation alone.^4^ Durvalumab was the first therapeutic in several decades to demonstrate a significant survival advantage compared with chemoradiation alone in this context.^3^ As such, durvalumab uptake has been rapid globally and now represents the new standard of care in this setting. Despite this practice change, many unanswered questions remain that are critical to improve survival in stage III NSCLC.

Criteria for clinical trial eligibility are often stringent and not reflective of real‐world patient cohorts, raising questions about the generalizability and appropriateness of using trial data in these settings. Studies examining real‐world patient cohorts with advanced lung cancer have shown that between 65% and 72% of patients are not eligible for clinical trials.^5^, ^6^ Subsets of patients including patients with advanced age, poor performance status, non‐white ethnicity, and certain comorbidities are particularly underrepresented in clinical trials but are commonly encountered in real‐world settings.^7^ Evaluating the use of durvalumab in routine clinical settings outside of the constraints of clinical trial criteria is important not only for the large subset of patients who are trial ineligible but to also understand the safety and efficacy profile of durvalumab in routine clinical practice.^7^, ^8^ In particular, patients were recruited to the PACIFIC trial at the conclusion of chemoradiation, therefore their fitness compared with standard clinical cohorts is critical.

We aimed to evaluate the proportion of stage III NSCLC patients eligible for durvalumab based on the PACIFIC trial at two Australian tertiary centers, the reasons for ineligibility, and assess the influence of eligibility and durvalumab use on patient outcomes to inform routine clinical practice.

## MATERIALS AND METHODS

### Study design and patients

Consecutive patients with stage III NSCLC treated at Westmead and Blacktown Hospitals in Sydney, Australia between January 2015 and July 2022 were included. All patients were discussed at the lung multidisciplinary team meeting and treatment decisions were made by consensus of the treating physicians.

Retrospective data were collected for patients who received definitive concurrent chemotherapy, defined as patients who received at least two cycles of combination platinum‐based chemotherapy with radiotherapy. We collected data on demographics, Eastern Cooperative Oncology Group (ECOG) performance status, tumor‐specific features (histologic subtype, stage as per the 8th edition of the American Joint Committee on Cancer Staging Manual), oncogenic alterations (*EGFR, ALK, ROS‐1, KRAS*), programmed death‐ligand 1 (PD‐L1) expression, and treatment details (radiotherapy dose, systemic therapy, consolidation durvalumab). Standard dose radiotherapy was defined as 60 Gray (Gy) in 2.0 size fractions as per the eligibility criteria of the PACIFIC trial.^3^, ^9^ Further details of radiotherapy treatment are outlined in Table S2. Induction chemotherapy prior to concurrent chemoradiotherapy was permitted. Patients were excluded if they received either chemotherapy or radiotherapy with palliative intent.

Best response to treatment was evaluated as per the Response Evaluation Criteria in Solid Tumors (RECIST), version 1.1. PFS was defined as the time from completion of radiotherapy to disease progression as per RECIST criteria or death. OS was defined as the time from completion of radiotherapy to death of any cause up to December 1, 2022.

Patients were defined as eligible for the PACIFIC trial if they fulfilled the inclusion criteria as specified in the clinical trial protocol (Table S1). Given the retrospective nature of this study, we did not specifically evaluate the exclusion criteria of prolonged QTc.^3^

We collected data on reasons for trial ineligibility. For all patients who received durvalumab, we collected data on early cessation of therapy, hospitalization or corticosteroid use for treatment‐related toxicity.

### Statistical analysis

Baseline characteristics between eligible and ineligible groups were compared using chi‐square tests and Student's *t*‐tests as appropriate. The Kaplan–Meier method was used to estimate PFS and OS. The log rank test was used to compare groups. The statistical analysis was performed using SPSS, version 27 (IBM Corp.). Univariate and multivariate analysis of variables associated with PFS and OS were performed using the Cox regression proportional hazard model with R version 4.2.1 for Windows. Univariate variables with a *p* value <0.20 were included in the multivariate model. For all analyses, a *p* value of <0.05 was considered statistically significant. The cut‐off date for all data used in the analyses was December 1, 2022.

The study was approved by the local Human Research Ethics Committee and a waiver of consent was granted given the retrospective nature of this study.

## RESULTS

**FIGURE 1:**
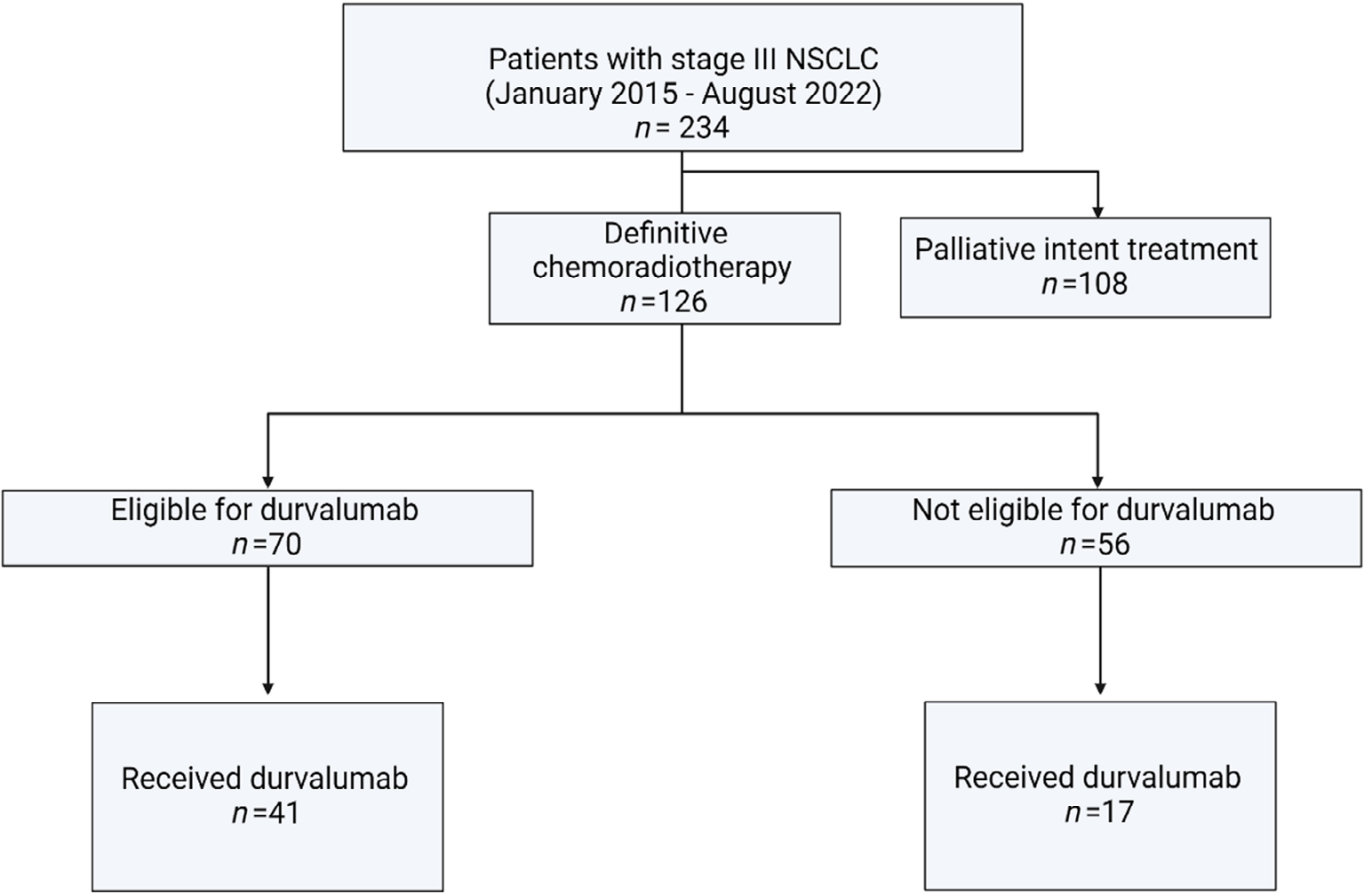
Study flowchart.

Figure 1 summarizes the study flowchart.

### Baseline patient characteristics

We identified 234 patients with stage III NSCLC; 126 received definitive concurrent chemoradiotherapy and were included for further analysis. Baseline characteristics are summarized in Table 1.

**TABLE 1:** Baseline patient characteristics for those with unresectable stage III non‐small‐cell lung cancer who proceeded with definitive chemoradiotherapy, including baseline characteristics of patients from the PACIFIC study, derived from Antonia et al.^3^

Characteristic	Durvalumab ineligible (*n* = 56)	Durvalumab eligible (*n* = 70)	PACIFIC (*n* = 713)^3^	Total (*n* = 126)	*p* value
Age, years					0.16
Median (range)	68 (49–83)	71 (50–85)	64 (23–90)	69 (49–85)	
Sex, *n* (%)					0.623
Male	36 (64)	42 (60)	500 (70)	78 (62)	
Female	20 (36)	28 (40)	213 (30)	48 (56)	
Disease stage, *n* (%)					0.264
IIIA	25 (45)	37 (53)	125 (53)	62 (49)	
IIIB	22 (39)	28 (40)	107 (45)	50 (40)	
IIIC	9 (16)	5 (7)	NA	14 (11)	
ECOG performance status, *n* (%)					0.424
0	26 (46)	39 (56)	114 (48)	65 (52)	
1	29 (52)	31 (44)	122 (52)	60 (48)	
2	1 (2)	0	0	1 (0.08)	
Tumor histologic type, *n* (%)					0.806
Squamous	22 (30)	26 (37)	102 (43)	48 (38)	
Non‐squamous	26 (46)	44 (63)	135 (57)	78 (62)	
Smoking status, *n* (%)					0.262
Current or former smoker	48 (86)	65 (93)	216 (91)	113 (90)	
Never smoked	8 (14)	5 (7)	21 (9)	13 (10)	
Radiotherapy, *n* (%)					
<54 Gy	10 (18)	2 (3)	0	12 (10)	0.004*
≥54 to ≤66 Gy	46 (82)	68 (97)	217 (92)	114 (90)	
Previous chemotherapy, *n* (%)					
Induction	2 (4)	2 (3)	68 (28)	4 (3)	
Concurrent with radiation therapy	56 (100)	70 (100)	236 (99.6)	126 (100)	
Best response to chemoradiotherapy, *n* (%)					
Complete response	0	1 (1)	7 (3)	1 (0.08)	<0.001*
Partial response	26 (46)	42 (60)	111 (47)	68 (54)	
Stable disease	14 (25)	26 (37)	114 (48)	40 (32)	
Progressive disease	13 (23)	0	0	13 (10)	
Not assessed	3 (5)	1 (1)	0	4 (3)	
Driver mutation, *n* (%)					
No driver mutation	48 (86)	60 (86)	Not reported	108 (86)	1.00
Driver mutation	8 (14)	10 (14)	Not reported	18 (14)	
Concurrent chemotherapy regimen, *n* (%)					0.087
Cisplatin/etoposide	12 (21)	24 (34)	155 (22)	36 (29)	
Carboplatin/paclitaxel	39 (70)	46 (66)	242 (34)	85 (67)	
Cisplatin/pemetrexed	1 (2)	0	16 (2)	1 (0.08)	
Unknown or other	4 (7)	0	300 (42)	4 (3)	
PD‐L1, *n* (%)					0.211
<1%	12 (21)	16 (23)		28 (22)	
1–49%	10 (18)	12 (17)	<25%: 187 (39)	22 (17)	
≥50%	14 (25)	8 (11)	≥25%: 115 (24)	22 (17)	
Unknown	20 (36)	34 (49)	174 (37)	54 (43)	
Durvalumab treatment, *n* (%)					0.001*
Yes	17 (30)	41 (59)	476 (67)	58 (46)	
PACIFIC (placebo or durvalumab)	0	3 (4)	NA	3 (2)	
No	39 (70)	26 (37)	213 (33)	65 (52)	
Completed 12 months durvalumab treatment, *n* (%)					
Yes	16 (94)	33 (80)	202 (42)	49 (84)	
No	1 (6)	8 (20)	274 (58)	9 (16)	
Mean time to durvalumab commencement	55 days	52 days	Not reported	58	0.554

Abbreviations: ECOG, Eastern Cooperative Oncology Group; NA, not applicable; PD‐L1, Programmed death‐ligand 1.

*Note*: Statistical analyses conducted between durvalumab eligible and ineligible groups, *p* < 0.05.

Most patients were male (62%) and current or former smokers (90%) with a median age of 69 years (range 49–85 years). ECOG performance status was 0–1 in 99% of patients. Most tumors were non‐squamous histology (62%) with no driver mutation (86%). Seven patients with EGFR mutations were included in both the eligible (10%) and ineligible (13%) cohorts. Four patients had induction chemotherapy. All patients received platinum doublet chemotherapy concurrently with radiotherapy. Recommended dose radiotherapy was delivered in 90% of patients.

Fifty‐eight patients (46%) received durvalumab treatment. Forty‐nine (84%) of these patients completed 12 months of treatment. The mean time to commence durvalumab following radiotherapy completion was 53 days (interquartile range 39–61 days).

### Eligibility for PACIFIC

Seventy patients (56%) were eligible for the PACIFIC trial. Fifty‐six patients (44%) did not meet eligibility criteria for the PACIFIC trial. Ineligibility reasons are summarized in Table 2. Most patients were ineligible due to history of previous malignancy (*n* = 18, 32%), progressive disease or death (*n* = 14, 25%) or receiving a radiotherapy dose <54 Gy (*n* = 9, 16%). Nine patients (18%) were ineligible due to clinically significant radiation pneumonitis. Five patients (9%) were ineligible due to pre‐existing autoimmune disease. Twelve patients (21%) had multiple reasons for ineligibility.

**TABLE 2:** Summary of reasons for PACIFIC trial ineligibility

Reason for PACIFIC trial ineligibility	*n* (%)	Received durvalumab (*n*)
Previous malignancy	18 (32)	14
Progressive disease or death	14 (25)	0
Radiotherapy dose <54 Gy	9 (16)	0
Radiation pneumonitis (CTCAE grade ≥2)	8 (14)	2
Best response not assessed	2 (4)	0
ECOG 2 or 3	3 (5)	1
History of autoimmune disorder or previous exposure to immunotherapy	5 (9)	0
Medical comorbidities	5 (9)	0
Consolidation chemotherapy	1 (2)	0
Multiple reasons	12 (21)	0

Abbreviations: CTCAE, Common Terminology Criteria for Adverse Events; ECOG, Eastern Co‐operative Oncology Group performance status.

### Outcomes

**FIGURE 2:**
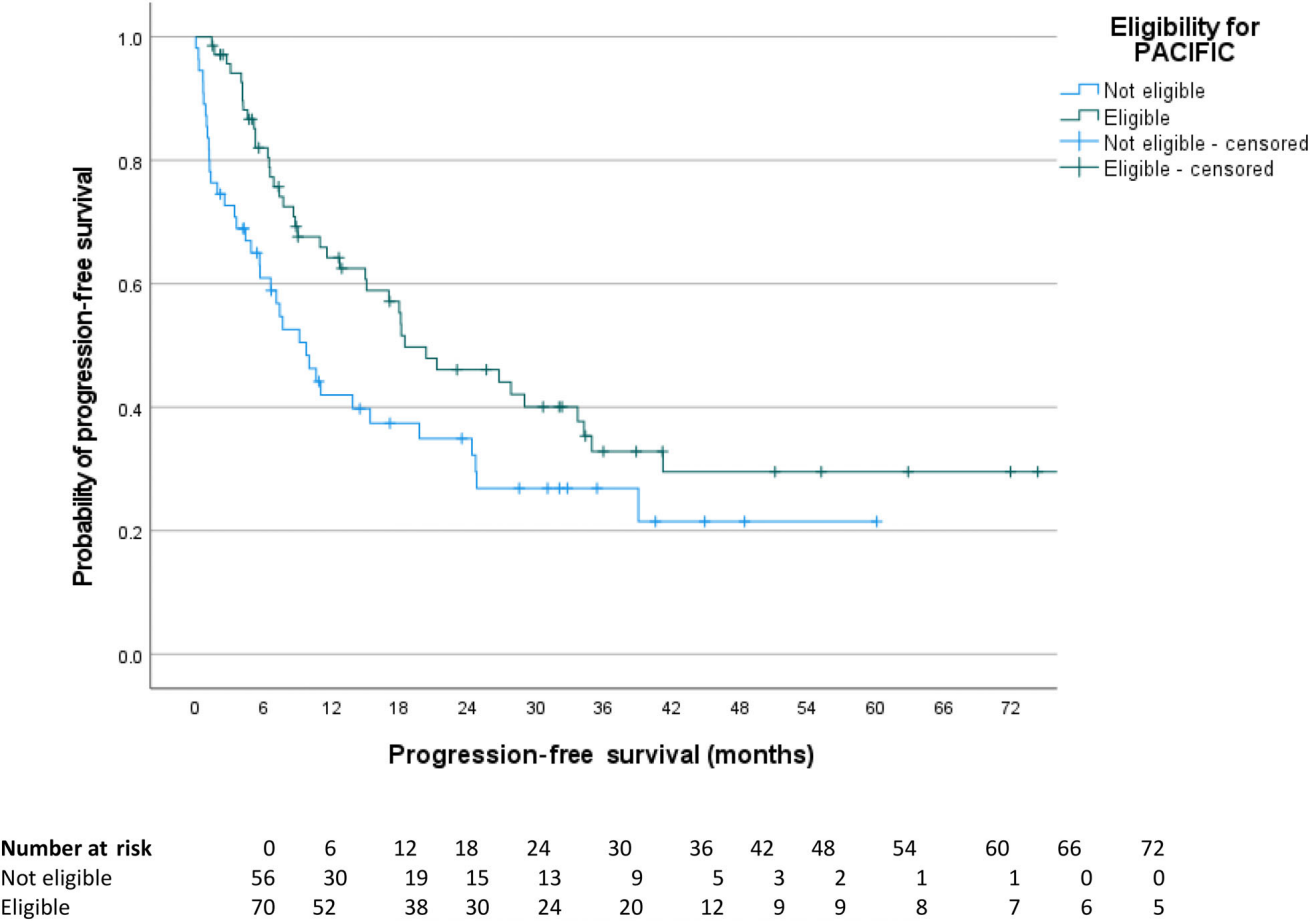
Kaplan–Meier curves of progression‐free survival as per the response evaluation criteria in solid tumors, version 1.1, in patients eligible (median 9.7 months) and ineligible (median 18.4 months) for the PACIFIC trial. Hazard ratio 0.61 (95% confidence interval 0.39–0.95), *p* = 0.029

**FIGURE 3:**
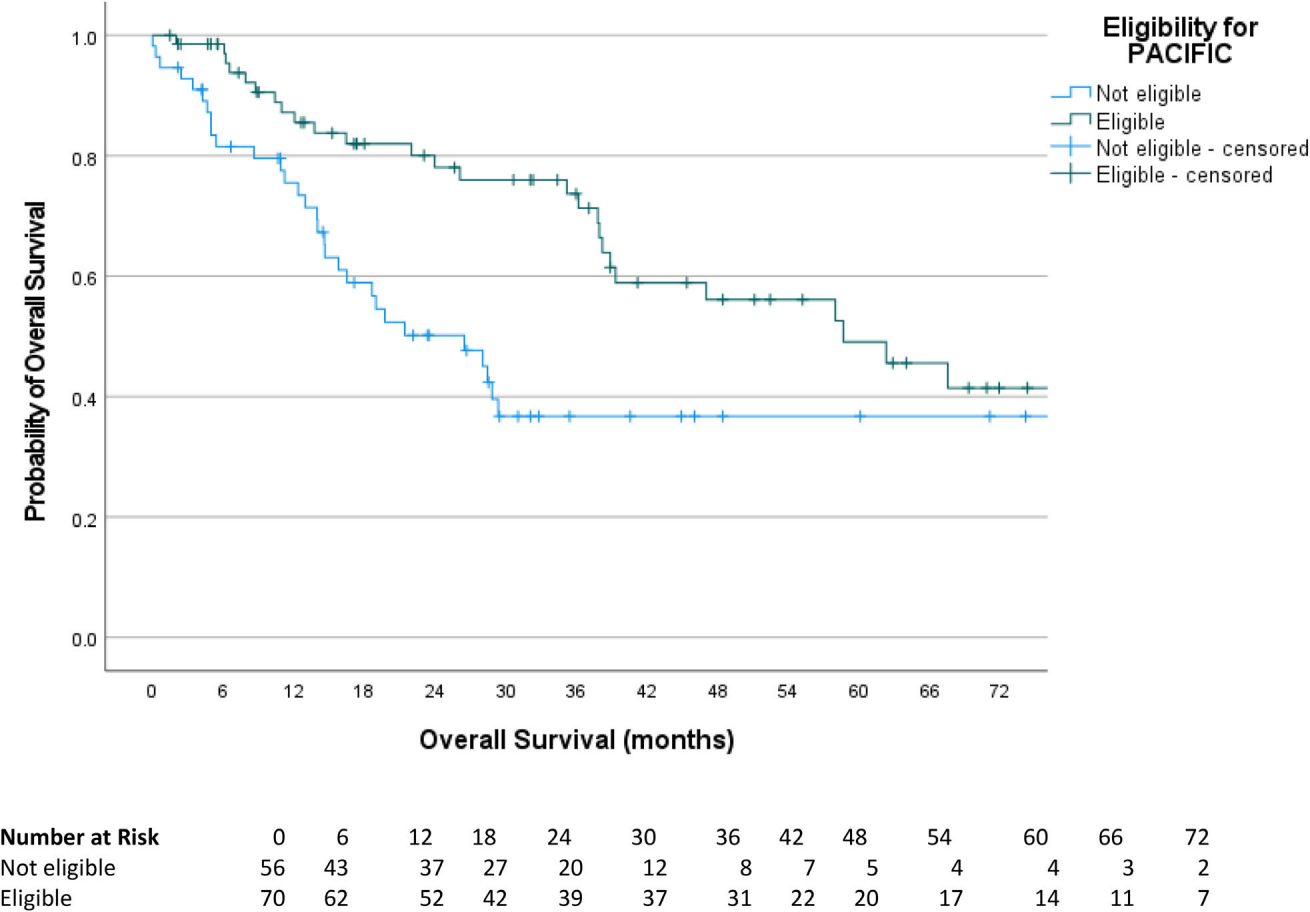
Kaplan–Meier curves of overall survival as per the response evaluation criteria in solid tumors, version 1.1, in patients ineligible (median 26.5 months) and eligible (median 58.7 months for the PACIFIC trial. Hazard ratio 0.47 (95% confidence interval 0.28–0.80), *p* = 0.005

Regardless of the receipt of durvalumab, the median PFS in all eligible patients was 18.4 months compared to 9.7 months in ineligible patients (hazard ratio [HR] 0.61, 95% confidence interval [CI] 0.39–0.95, log rank *p* = 0.029) (Figure 2). The median OS in all eligible patients regardless of receipt of durvalumab was 58.7 months compared to 26.5 months in ineligible patients (HR 0.47, 95% CI 0.28–0.80, log rank *p* = 0.005) (Figure 3).

When patients that had progressive disease after chemoradiotherapy regardless of receipt of durvalumab were not analyzed in the survival outcome, median OS remained significantly shorter in all ineligible patients compared to eligible patients (28.4 vs. 58.7 months, HR 0.51, 95% CI 0.28–0.95, log rank *p* = 0.03; Figure S1). PFS was also shorter but did not reach statistical significance (15.4 vs. 20.3 months, HR 0.92, 95% CI 0.54–1.55, log rank *p* = 0.75; Figure S2).

**FIGURE 4:**
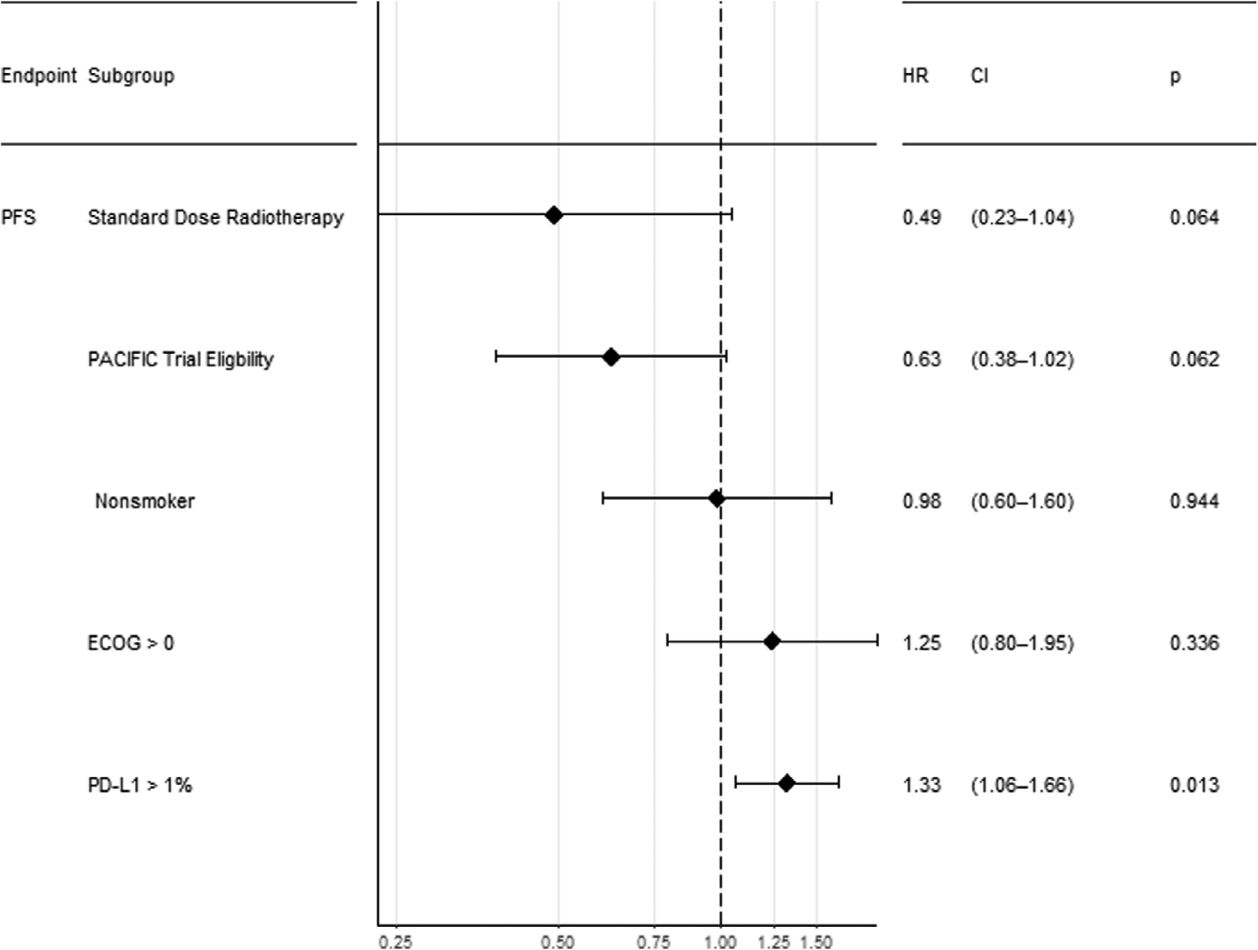
Multivariable Cox proportional hazards analysis showing hazard ratios for progression‐free survival for Eastern Co‐operative Oncology Group, PACIFIC trial ineligibility, radiotherapy dose, smoking status, and histology in the overall cohort. *p < 0.05

**FIGURE 5:**
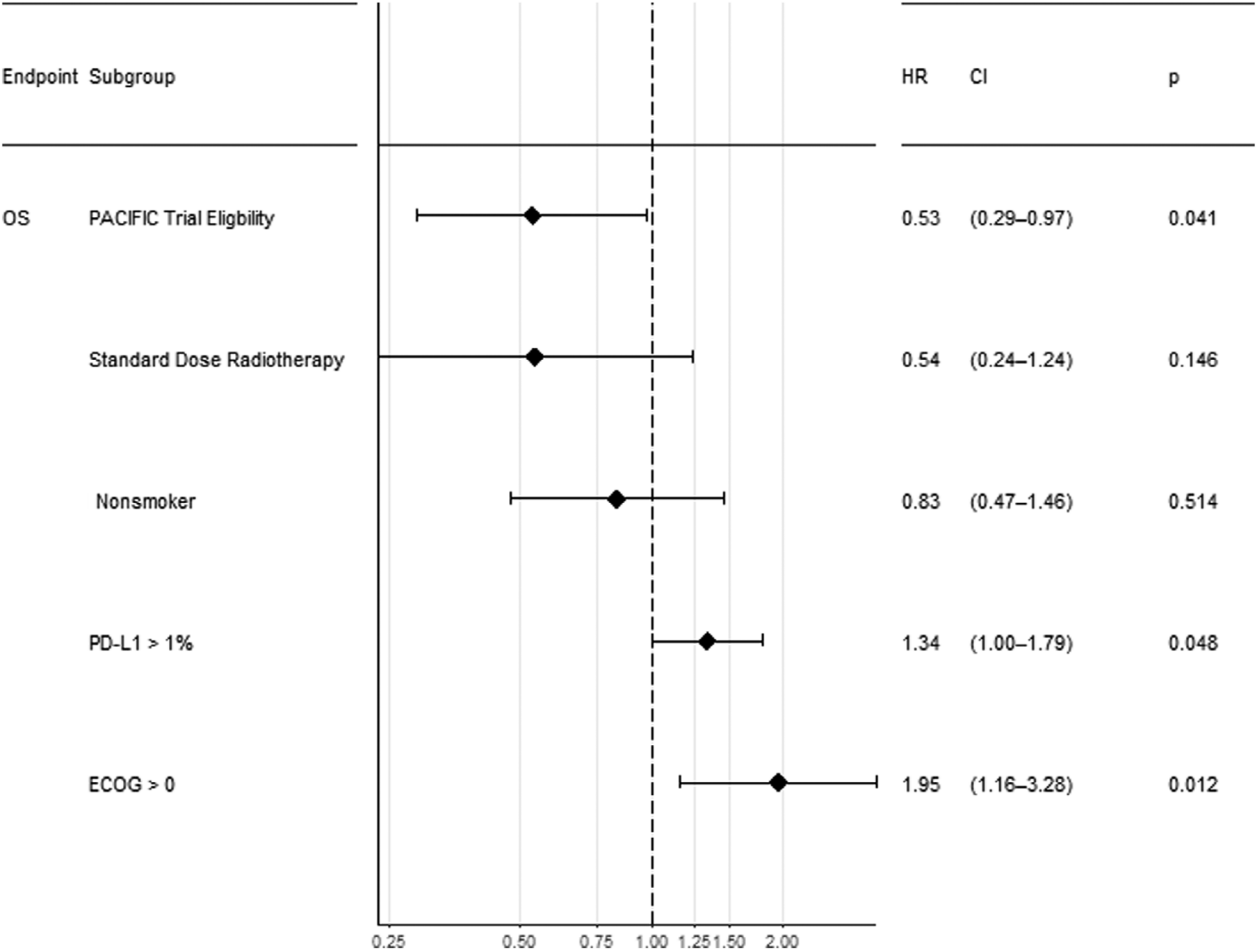
Multivariable Cox proportional hazards analysis showing hazard ratios for overall survival for Eastern Co‐operative Oncology Group, PACIFIC trial ineligibility, less than standard dose radiotherapy, smoking status, and histology in the overall cohort. *p < 0.05

In univariate analysis, patients who received less than standard dose radiotherapy had shorter PFS (HR 3.38, 95% CI 1.71–6.68, *p <* 0.001) and OS (HR 3.44, 95% CI 1.68–7.02, *p* < 0.001) (Figure S3). PACIFIC trial eligibility (HR 0.6, 95% CI 0.38–0.93, *p* = 0.006) and ECOG performance status 0 (HR 0.46, 95% CI 0.27–0.78, *p* = 0.004) were associated with a significant improvement in OS (Table 3). In the 72 patients with available data on PD‐L1 staining, there was a trend to improved survival outcomes with lower PD‐L1 expression overall (Figure S4). In multivariate analysis, receiving a standard dose of radiotherapy (HR 0.49, 95% CI 0.23–1.04, *p* = 0.06) and PACIFIC trial eligibility (HR 0.63, 95% CI 0.38–1.02, *p* = 0.06) remained associated with improved PFS (Figure 4). The only variable significantly associated with improved OS in the multivariate model was PACIFIC trial eligibility (HR 0.53, 95% CI 0.29–0.97, *p* = 0.04; Figure 5).

**TABLE 3:** Univariate Cox proportional hazards regression analysis showing hazard ratios for progression‐free survival and overall survival for different variables in the overall cohort

Variable	Progression‐free survival			Overall survival		
	HR	95% CI	*p* value	HR	95% CI	*p* value
PACIFIC Eligibility	0.47	0.28–0.8	0.006*	0.60	0.38–0.93	0.006*
Age <65 years	0.95	0.6–152	0.832	0.91	0.53–1.58	0.749
Stage IIIA	0.93	0.59–1.46	0.752	1.05	0.62–1.78	0.855
Squamous histology	0.34	0.55–0.90	0.016*	0.93	0.54–1.6	0.802
Driver mutation	0.62	0.35–1.09	0.095	1.21	0.56–2.58	0.63
Nonsmoker	0.91	1.43–2.24	0.12	1.6	0.94–2.72	0.086
Male sex	0.95	0.60–1.51	0.842	1.06	0.61–1.82	0.842
ECOG 0	0.74	0.47–1.16	0.19	0.46	0.27–0.78	0.004*
PD‐L1						
<1% vs. 1–49%	0.73	0.31–1.71	0.463	0.28	0.07–1.06	0.061
<1% vs. ≥50%	0.37	0.17–0.82	0.015*	0.18	0.05–0.64	0.008*
Less than standard radiotherapy dose	3.38	1.71–6.68	<0.001*	3.42	1.67–7.02	<0.001*
Cisplatin/etoposide chemotherapy	1.13	0.7–1.83	0.601	0.73	0.4–1.32	0.296
Time to durvalumab commencement <42 days	1.24	0.51–3.03	0.64	1.07	0.33–3.43	0.913

*Note*: *p* < 0.05.

### Analysis of patients who received durvalumab

#### Ineligible patients

Seventeen ineligible patients (30%) received durvalumab. Fourteen of these patients were ineligible due to a history of previous malignancy (*n* = 4 head and neck cancer, *n* = 2 cervical cancer, *n* = 1 liposarcoma, *n* = 1 melanoma, *n* = 1 breast cancer, *n* = 1 bladder carcinoma, *n* = 1 lymphoma, *n* = 1 second primary NSCLC, *n* = 2 colorectal cancer). One patient was ineligible due to ECOG 2 performance status post chemoradiotherapy. For patients who were ineligible for the PACIFIC trial but received durvalumab treatment, there was no significant difference in median PFS compared with eligible patients (median not reached vs. 26.8 months, *p* = 0.43). Median OS was not reached in either group (*p* = 0.80).

Two ineligible patients ceased durvalumab early (*n* = 1 due to declining ECOG, *n* = 1 due to pneumonitis) but otherwise there was no early cessation of therapy, hospitalizations, immune‐related endocrinopathies, or corticosteroid use for treatment‐related toxicity in this patient group.

#### Eligible patients

Forty‐one (60%) trial‐eligible patients received durvalumab following approval and availability in our jurisdiction. Median PFS in eligible patients who did not receive durvalumab was 8.8 months compared to 26.8 months in eligible patients who received durvalumab (HR 0.63, 95% CI 0.36–1.10, *p* = 0.10) (Figure S5). Median OS in eligible patients who did not receive durvalumab was 58.7 months and not reached in eligible patients who received durvalumab (HR 0.91, 95% CI 0.49–1.70, *p =* 0.77) (Figure S6). Seven patients developed treatment‐related toxicity in this group requiring corticosteroid therapy. Two patients were hospitalized (*n* = 1 for suspected autoimmune nephritis, *n* = 1 for pneumonitis; Table S3).

## DISCUSSION

In this study, we evaluated the eligibility of patients with stage III NSCLC for the PACIFIC trial, reasons for ineligibility, and the impact on outcomes in a real‐world setting at two Australian tertiary hospitals. There were several key findings. First, we noted that a significant proportion of our patient cohort who proceeded to definitive chemoradiotherapy (44%) were ineligible for the PACIFIC trial. Second, trial eligibility resulted in improved outcome regardless of receipt of durvalumab. Third, a subset of ineligible patients who received durvalumab had similar outcomes to trial‐eligible patients who received durvalumab.

Our study demonstrated that only 56% of patients treated with definitive chemoradiotherapy and 30% of all patients with stage III NSCLC were eligible for the PACIFIC trial. This data is in keeping with a previous retrospective cohort series of definitive chemoradiation patients in Japan, Canada, Korea, and Germany which found that 65%, 60%, 56%, and 50% of patients, respectively, were eligible for durvalumab.^10^, ^11^, ^12^, ^13^ An additional Japanese study which only examined radiation pneumonitis, poor performance status, and disease progression found that 70% of patients were eligible to receive durvalumab.^14^ In contrast, a total of 75% of patients that were screened ultimately enrolled in the PACIFIC study, suggesting that the study population was preselected.^3^

This has given rise to concern about the generalizability of the PACIFIC trial results to real‐world populations, including our cohort. Compared to the PACIFIC study, in our cohort, patients were older and mostly commenced durvalumab treatment >42 days following the completion of chemoradiotherapy. In the PACIFIC study, PFS and OS were defined as time from randomization, which could occur up to 42 days following completion of radiotherapy. Although in our study, PFS and OS were defined as the time from completion of radiotherapy, the survival outcomes compare favorably to the results from this study.^4^ These data add to the increasing body of real‐world evidence confirming the benefits of durvalumab use in real‐world patient populations.^13^, ^15^, ^16^ Similarly, to our cohort, a recent meta‐analysis of 1885 patients found that in the real world, patients treated with durvalumab were older, commenced durvalumab >42 days following completion of chemoradiotherapy and had worse performance status. Despite this, as in our cohort, durvalumab use appeared to be safe and beneficial in patients who received treatment.^7^

Patients ineligible for the PACIFIC trial had shorter median PFS (9.7 months) and OS (18.4 months). The detrimental effect on OS was maintained even when patients with progressive disease were excluded (*p* = 0.005). PACIFIC trial eligibility was also associated with improved outcomes in both univariate and multivariate analysis. This supports previous studies which have demonstrated that trial eligibility is a positive prognostic factor, including a previous case series which specifically looked at PACIFIC trial eligibility.^10^, ^17^, ^18^, ^19^, ^20^ The most common reasons for PACIFIC trial ineligibility in our cohort were history of a previous malignancy (32%), progressive disease or death during chemoradiotherapy (25%), receiving a radiotherapy dose <54 Gy (16%), and radiation pneumonitis (14%). In contrast, the most common reasons for ineligibility in the German and Japanese retrospective cohorts were progressive disease or death.^10^, ^12^ In the Korean cohort, the most common reasons for ineligibility were related to radiotherapy dose or pneumonitis.^13^

Despite ineligibility for the PACIFIC trial, 17 patients (30%) received durvalumab treatment. Most of these patients were ineligible due to history of another malignancy. In this small group of patients there was no detrimental impact on outcomes seen when compared to trial‐eligible patients who received durvalumab. Only two patients ceased treatment early, one due to declining performance status and one due to pneumonitis. Few studies have evaluated the impact of cancer treatment among trial‐eligible patients. In a Korean study in which 56% of patients were ineligible for the PACIFIC trial, mostly related to radiotherapy dose, favorable PFS was demonstrated with durvalumab use in the ineligible patients (PFS not reached vs. 9.6 months, *p* = 0.06). A large Canadian study of over 125 000 patients across multiple tumor streams found that ineligible patients had worse outcomes than trial eligible patients, but when these patients received treatment, they had improved outcomes compared to patients who did not receive any treatment.^19^ A subset of patients in our cohort (*n* = 5) were ineligible based on the presence of autoimmune disorders. Most of these patients had a diagnosis of rheumatoid arthritis, which may not be an absolute contraindication to receiving immunotherapy in standard practice.^21^ Collectively, these data suggest that durvalumab use following chemoradiotherapy may be beneficial and safe in certain subsets of trial ineligible patients.

In univariate analysis, in addition to trial eligibility, the strongest factor associated with survival outcome was receipt of radiation dose <54 Gy. Multivariate regression analysis demonstrated a trend to worse outcomes with receipt of radiation dose <54 Gy. In the RTOG 7301 study, compared to lower doses of radiotherapy, 60 Gy in 30 daily fractions over a 6‐week period was associated with improved tumor response.^9^ In practice, the use of a radiation dose <54 Gy may reflect large tumor volume and technical inability to deliver the full radiotherapy dose. Large tumor volume has been associated with poor prognosis in several studies.^22^, ^23^

Unexpectedly, univariate and multivariate regression analysis demonstrated improved PFS and OS with negative PD‐L1‐expression. The PACIFIC trial was not designed or powered to evaluate the effect of PD‐L1 expression on outcome, but post hoc analyses demonstrated that there was a significant improvement in all subgroups except for patients with PD‐L1 < 1%.^24^ Published data show conflicting findings, with some data indicating PD‐L1 expression may be associated with worse survival.^25^, ^26^ Another study suggested that PD‐L1 positivity may confer an improved prognosis in squamous cell NSCLC.^27^ There is limited data on the value of PD‐L1 expression in stage III NSCLC patients treated with concurrent chemoradiotherapy. One study suggests that there is a trend for worse survival with PD‐L1 expression, particularly in association with low density of CD8+ tumor infiltrating lymphocyte density.^28^ In contrast, a multicentre retrospective analysis of 147 Canadian patients found that patients with PD‐L1 expression >50% benefitted most from durvalumab.^29^ Conclusions from our cohort are limited by the large number of patients (*n* = 54) who had unknown levels of PD‐L1 expression. Larger series are required to examine the predictive and prognostic roles of PD‐L1 expression in stage III NSCLC.

The strengths of this study are that it provides a real‐world description of a cohort with stage III NSCLC and outcomes in two Australian tertiary centres and adds to the increasing body of real‐world evidence in this space. Although we agree with previous studies that trial eligibility criteria should be broadened to reflect routine clinical practice, in the absence of this, real‐world data can help to bridge this gap. We confirm that trial eligibility is associated with improved prognosis in keeping with several other real‐world studies.^10^, ^19^ Furthermore, we provide insight into subsets of patients who may benefit from durvalumab regardless of trial ineligibility. Limitations include the retrospective design with the inherent risk of selection bias as well as the small subgroup numbers in several analyses. The follow‐up data period for patients who received durvalumab is also short, with most patients having less than 2 years of follow‐up data available.

## CONCLUSIONS

Only 56% of patients treated at our centres with concurrent chemoradiotherapy for stage III NSCLC met the eligibility criteria for the PACIFIC trial. Trial‐eligible patients had improved prognosis irrespective of durvalumab. A subset of ineligible patients who received durvalumab did not have worse outcomes or increased adverse events compared to trial‐eligible patients. The decision to proceed with durvalumab treatment in patients following chemoradiation should be individualized. Further studies are needed to explore the relationship of PD‐L1 expression and benefit from durvalumab.

## AUTHOR CONTRIBUTIONS

E.B. and A.N. conceptualized the manuscript and methodology. E.B. wrote the original draft manuscript and was responsible for data curation and formal analysis under the supervision of A.N. A.N., B.G., R.H., I.D.S., E.H., and H.G reviewed and edited the manuscript. All authors have read and agreed to the published version of the manuscript.

## FUNDING INFORMATION

This research received no external funding. E.B. is supported by the Royal Australasian College of Physicians Arnott Research Entry Scholarship in Cancer Research (2022).

## CONFLICT OF INTEREST

There are no conflicts of interest. See below for author disclosures: I.D.S.: Consultant Advisor for MSD; Speaker honoraria: Roche, Novartis, Bristol Myers Squibb. RH.: Advisory board member for AstraZeneca, BMS, Eisai, Eli Lilly, Merck, MSD, Novartis, Oncosec, Pfizer, Roche, Seagen; Speaker Honorarium from AstraZeneca, MSD, Novartis, Roche. A.N.: Advisory board member for MSD, BMS, Roche, Astra Zeneca, PfizerMerck Serono.

## INSTITUTIONAL REVIEW BOARD STATEMENT

The study was conducted according to the guidelines of the Declaration of Helsinki and approved by the Western Sydney Local Health District Human Research Ethics Committee (Ethics reference: 2021/ETH00955, date of approval 9 August 2021).

## INFORMED CONSENT STATEMENT

Patient consent was waived given the retrospective nature of this study.

## Supporting information


**Data S1:** Supporting Information
