# Supplementary material for: Use of durvalumab in stage III non‐small‐cell lung cancer based on eligibility for the PACIFIC study
Source: Thorac Cancer. 2023 Jan 10;14(6):563–72. doi: 10.1111/1759-7714.14780 (PMC9968599; doi:10.1111/1759-7714.14780)
Supplement: Supplementary file 1 — Data S1: Supporting Information [file TCA-14-563-s001.docx]

**Supplementary Material**

**Supplementary Tables**

| **Inclusion Criteria** | **Exclusion Criteria** |
| --- | --- |
| Male or female aged 18 years or older | Mixed small cell and non-small cell histology |
| Histologically confirmed non-small cell lung cancer with locally advanced, unresectable (stage III) disease | Patients who received sequential chemoradiation therapy |
| Received at least 2 cycles of platinum-based chemotherapy concurrent with radiation therapy | Patients who have progressed during or immediately following (within 6 weeks) of definitive concurrent chemoradiation |
| Chemotherapy regimen must include one of either etoposide, vinblastine, vinorelbine, a taxane or pemetrexed | Prior exposure to any anti-programmed death-1 or anti-programmed death-ligand 1 antibody |
| Consolidation chemotherapy following radiation not allowed but chemotherapy prior to concurrent chemoradiation is allowed | Current or prior use of immunosuppressive medication within 28 days before the first dose of durvalumab (except for inhaled corticosteroids and systemic corticosteroids at physiological doses) |
| Patients must have received a total dose of radiation of 60 Gray (Gy) +/- 10% (54Gy to 66 Gy) | Patients with ≥ grade 2 pneumonitis |
| ECOG performance status of 0 or 1 | Active or prior documented autoimmune disease within the past 2 years, history of primary immunodeficiency, history of allogeneic organ transplant |
| Adequate organ and bone marrow function as defined by:   - Absolute neutrophil count > 1.5 x 10^9/L - Platelets > 100 x 10^9/L - Haemoglobin ≥ 9.0 g/dL - Serum creatinine clearance > 40ml/min by the Cockcroft-Gault formula - Serum bilirubin ≤ 1.5 x upper limit of normal - AST and ALT ≤ 2.5 x upper limit of normal | Uncontrolled intercurrent illness including ongoing or active infection, symptomatic congestive heart failure, active bleeding diatheses, or psychiatric or social situations limiting compliance with follow up |
|  | History of previous malignancy treated in the last 5 years |

**Table S1 –** Modified inclusion and exclusion criteria used to evaluate durvalumab eligibility following chemoradiation for the study. Criteria modified from the PACIFIC trial (3).

Patients are simulated with a 4D-CT with PET/CT fusion and IV contrast if there is nodal involvement. The iGTV is delineated using on all bins of the respiratory cycle and expanded to an ITV with a 5-8mm margin taking into account normal structures. A 5mm PTV expansion is then utilized. Most patients are planned using either a volumetric or intensity modulated radiotherapy technique. The target dose is 60Gy in 30 fractions though if dosimetric constraints are not met due to tumor size or location the doses may be reduced to 54Gy in 27 fractions. The target coverage is 98% of PTV covered by the 95% prescribed dose. Normal tissue constraints include the spinal cord max, lung mean, V20, V30; esophagus and heart mean, V50 and V5.

**Table S2 -** Details of radiotherapy treatment in the patient cohort

| **Toxicity** | **n (%)** |
| --- | --- |
| Hypothyroidism | 4 (7%) |
| Pneumonitis | 6 (10%) |
| Nephritis | 1 (2%) |
| Diabetes mellitus | 1 (2%) |
| Neurotoxicity | 1 (2%) |
| Colitis | 1 (2%) |

**Table S3 –** Summary of immune related toxicities in patients treated with durvalumab (n=58 patients in total treated with durvalumab).

**Supplementary Figures**


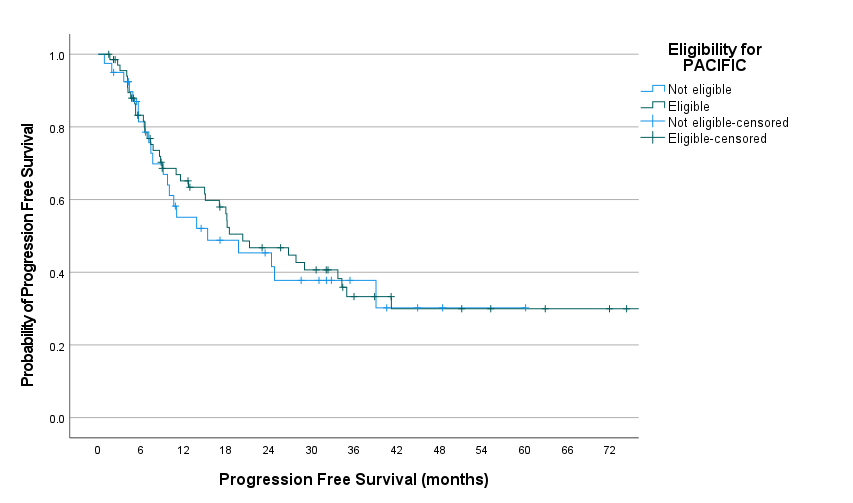


| **Number at Risk** | 0 | 6 | 12 | 18 | 24 | 30 | 36 | 42 | 48 | 54 | 60 | 66 | 72 |
| --- | --- | --- | --- | --- | --- | --- | --- | --- | --- | --- | --- | --- | --- |
| Not eligible | 39 | 29 | 18 | 14 | 12 | 9 | 5 | 3 | 2 | 1 | 1 | 0 | 0 |
| Eligible | 68 | 52 | 38 | 30 | 24 | 20 | 12 | 9 | 9 | 8 | 7 | 6 | 5 |

**Figure S1 –** Kaplan-Meier curves of progression free survival (patients with progressive disease excluded) as per the Response Evaluation Criteria in Solid Tumours, version 1.1, in patients ineligible (median 15.4 months) and eligible (median 20.3 months) for the PACIFIC trial with (hazard ratio 0.92, 95% confidence interval 0.54-1.55*, p=*0.75).


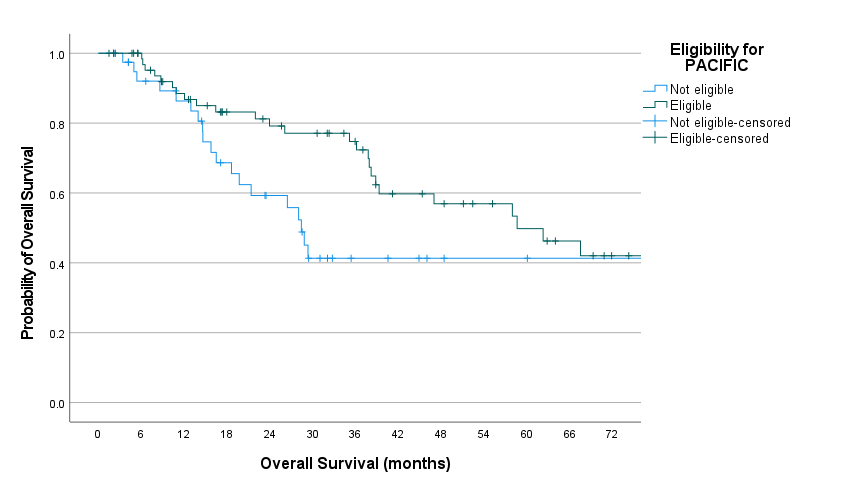


| **Number at Risk** | 0 | 6 | 12 | 18 | 24 | 30 | 36 | 42 | 48 | 54 | 60 | 66 | 72 |
| --- | --- | --- | --- | --- | --- | --- | --- | --- | --- | --- | --- | --- | --- |
| Not eligible | 39 | 34 | 30 | 22 | 17 | 10 | 6 | 5 | 3 | 2 | 2 | 1 | 1 |
| Eligible | 68 | 62 | 52 | 42 | 39 | 37 | 31 | 22 | 20 | 17 | 14 | 11 | 7 |

**Figure S2:** Kaplan-Meier curves of overall survival (patients with progressive disease excluded) as per the Response Evaluation Criteria in Solid Tumours, version 1.1, in patients ineligible (median 28.4 months) and eligible (median 58.7 months) for the PACIFIC trial (hazard ratio 0.51, 95% confidence interval 0.28-0.95, *p*=0.031).


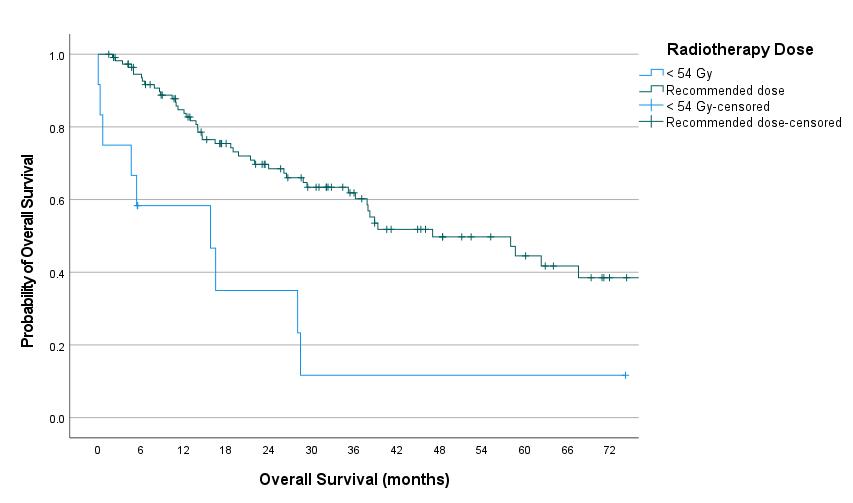


| **Number at Risk** | 0 | 6 | 12 | 18 | 24 | 30 | 36 | 42 | 48 | 54 | 60 | 66 | 72 |
| --- | --- | --- | --- | --- | --- | --- | --- | --- | --- | --- | --- | --- | --- |
| < 54 Gy | 11 | 4 | 4 | 3 | 3 | 1 | 1 | 1 | 1 | 1 | 1 | 1 | 1 |
| Recommended Dose | 113 | 100 | 84 | 66 | 56 | 48 | 38 | 28 | 24 | 20 | 17 | 13 | 8 |

**Figure S3 –** Kaplan-Meier curves of overall survival with patients as per the Response Evaluation Criteria in Solid Tumours, version 1.1, in patients who received < 54 Gy radiotherapy (median 15.8 months) and recommended dose radiotherapy (median 47.0 months), HR 0.29 (95% CI 0.14-0.60), log rank test *p* <0.001.


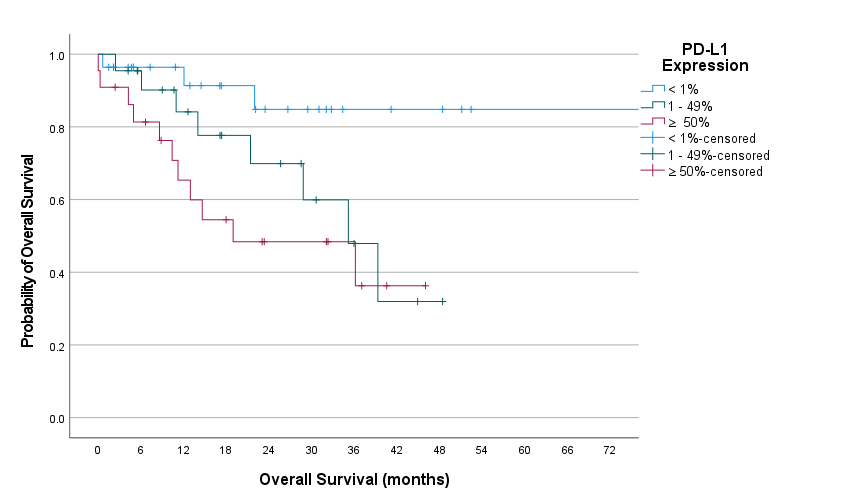


| **Number at Risk** | 0 | 6 | 12 | 18 | 24 | 30 | 36 | 42 | 48 | 54 | 60 | 66 | 72 |
| --- | --- | --- | --- | --- | --- | --- | --- | --- | --- | --- | --- | --- | --- |
| PD-L1 < 1% | 27 | 21 | 19 | 14 | 11 | 9 | 5 | 4 | 4 | 1 | 1 | 1 | 1 |
| PD-L1 1-49% | 21 | 18 | 14 | 10 | 9 | 6 | 3 | 2 | 1 | 0 | 0 | 0 | 0 |
| PD-L1 > 50% | 21 | 17 | 12 | 9 | 6 | 6 | 5 | 1 | 0 | 0 | 0 | 0 | 0 |

**Figure S4** – Kaplan-Meier curves of overall survival with patients as per the Response Evaluation Criteria in Solid Tumours, version 1.1, separated by PD-L1 expression status, log rank test *p*=0.02.


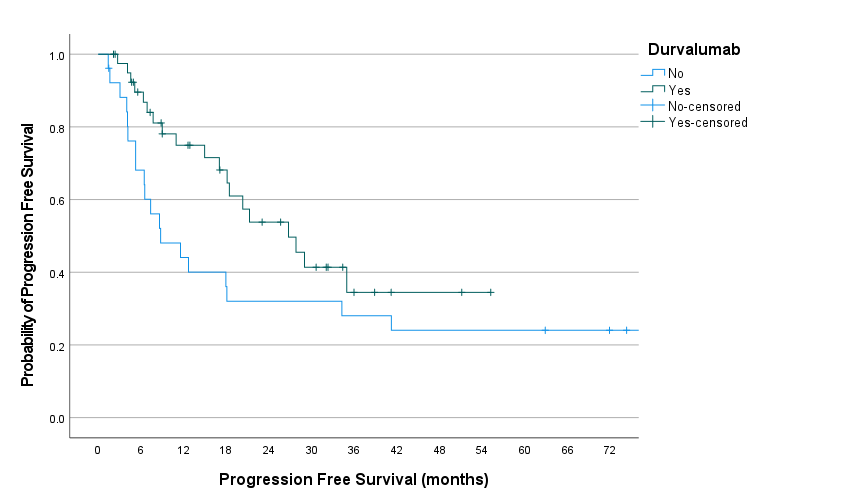


| **Number at Risk** | 0 | 6 | 12 | 18 | 24 | 30 | 36 | 42 | 48 | 54 | 60 | 66 | 72 |
| --- | --- | --- | --- | --- | --- | --- | --- | --- | --- | --- | --- | --- | --- |
| No durvalumab | 25 | 17 | 11 | 9 | 8 | 8 | 7 | 6 | 6 | 6 | 6 | 5 | 4 |
| Durvalumab | 40 | 32 | 24 | 19 | 14 | 10 | 4 | 2 | 2 | 1 | 0 | 0 | 0 |

**Figure S5** – Kaplan-Meier curves of progression free survival of eligible patients as per the Response Evaluation Criteria in Solid Tumours, version 1.1 who received durvalumab (median 26.8 months, 95% CI 1.9-15.6 months) and who did not receive durvalumab (median 8.8 months, 95% CI 17.0- 36.6 months), log rank test *p*=0.08.


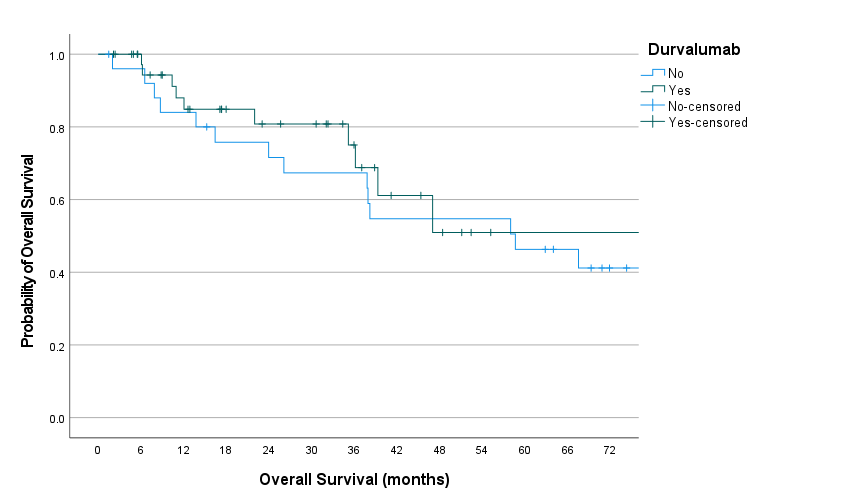


| **Number at Risk** | 0 | 6 | 12 | 18 | 24 | 30 | 36 | 42 | 48 | 54 | 60 | 66 | 72 |
| --- | --- | --- | --- | --- | --- | --- | --- | --- | --- | --- | --- | --- | --- |
| No durvalumab | 25 | 24 | 21 | 18 | 17 | 16 | 16 | 13 | 13 | 13 | 11 | 9 | 5 |
| Durvalumab | 40 | 35 | 28 | 21 | 19 | 18 | 12 | 7 | 5 | 2 | 1 | 1 | 1 |

**Figure S6** – Kaplan-Meier curves of overall survival of eligible patients as per the Response Evaluation Criteria in Solid Tumours, version 1.1 who received durvalumab (median not reached) and who did not receive durvalumab (median 58.7 months, 95% CI 27.9 - 88.2 months), log rank test *p*=0.65.
